# Formulation and *In vitro* Evaluation of Sustained Release Dosage Form with Taste Masking of Metformin Hydrochloride

**DOI:** 10.4103/0250-474X.65031

**Published:** 2010

**Authors:** P. K. Bhoyar, D. M. Biyani

**Affiliations:** S. K. B. College of Pharmacy, New Kamptee, Nagpur-441 002, India

**Keywords:** Hydroxypropylmethylcellulose, indion 244 and 264, metformin hydrochloride, sustained release, taste masking

## Abstract

An attempt was made to sustain the release of metformin HCl as well as to mask the bitter taste by complexation technique using strong cation-exchange resins, indion 244 and indion 264. The drug loading onto ion-exchange resin was optimized for mixing time, activation, effect of pH, mode of mixing, ratio of drug:resin and temperature. The resinate was evaluated for micromeritic properties, taste masking and characterized using XRPD and IR. Using resinate sustained release tablets were formulated using hydoxypropylmethylcellulose K100M.The tablets were evaluated for hardness, thickness, friability, drug content, weight variation and *in vitro* drug release. Tablets thus formulated (Batch B-6) provided sustained release of drug over a period of 10 h with first order kinetics. The release of metformin HCl from resinate controls the diffusion of drug molecules through the polymeric material into aqueous medium. Results showed that metformin HCl was successfully taste masked and formulated into a sustained dosage form as an alternative to the conventional tablet.

Ion exchange resinates (IER) have received considerable attention from pharmaceutical scientists because of their versatile properties as drug delivery vehicles. Ion exchange resins are cross-linked, water insoluble, polymer-carrying, ionizable functional groups. Drugs can be loaded onto the resins by an exchanging reaction, and hence, a drug-resin complex (drug resinate) is formed[1]. Ion exchange can be define as a reversible process in which ions of like sign are exchanged between liquid and solid, a highly insoluble body in contact with it[2]. The drug is released from the resinates by exchanging with ions in the gastrointestinal fluid, followed by drug diffusion[3]. Being high molecular weight water insoluble polymers, the resins are not absorbed by the body and are therefore inert.

During the past few years, IER have been extensively studied in the development of novel drug delivery system and other biomedical applications. Several IER products for oral and peroral administration have been developed for immediate release and sustained release purposes. Research over the last few years has revealed that IER are equally suitable for drug delivery technologies, including controlled release, transdermal, nasal, topical and taste masking[4]. Synthetic ion exchange resins have been used in pharmacy and medicine for taste masking or controlled release of drug as early as 1950[5–6]. Madgulkar evaluated sustained release behavior of venlafaxin resinates using response surface methodology[7].

Ion exchange resins have been increasingly used as taste masking agent[8]. The complex of cationic drug and strong cation exchange resin does not break at the pH of saliva i.e. 6-7 with cation concentration of 40 mEq/l, thereby imparting no bitter taste in the mouth. Mundada and Bhalekar[9] reduced the bitterness of roxithromycin using amberlite IRP 64 and Indion 214 resins. Venkatesh and Geetha Rao[10] reported taste masking of ambroxol hydrochloride with Indion 234 weak cation exchanger. In the present work strong cation exchange resins i.e. Indion 244 and Indion 264 were employed in order to get the sustained release profile of metformin hydrochloride.

## MATERIALS AND METHODS

Metformin hydrochloride–IP was a gift sample from Zim Laboratories (Nagpur, India). Indion 244 and Indion 264 were obtained from Ion Exchange India Ltd., Mumbai, India. MCC (PH 102) and HPMC (K100M) were obtained from Loba Chemie, Mumbai, India. All other chemicals and reagents used were of high analytical grade.

### Preparation of drug resin complex (Resinate):

Resinates were prepared by Batch process[11]. An accurately weighed amount of drug (0.1 g) was dissolved in 100 ml of distilled water. Then ion exchange resin (0.1 g) was added and stirred on a magnetic stirrer. Resinate thus formed was filtered and washed with copious amount of deionised water to remove any uncomplexed drug. It was then dried at 50° and the drug content was determined spectrophotometrically at 233.5 nm.

### Determination of drug content in the resinate:

An accurately weighed amount of drug equivalent resinate (0.1 g) was dissolved in 100 ml of 0.1N HCl and stirred for 5 h. Then the suspension was filtered, further dilutions were made and the drug content was determined at 233.5 nm using 0.1N HCl as a blank.

### Optimization of metformin hydrochloride-indion resin complexation:

The drug loading on to resin was optimized for various parameters such as mixing time, activation, effect of pH, mode of mixing, ratio of drug: resin and effect of temperature. Separate batches of indion 244 and indion 264 (0.1 g) were soaked in 100 ml of distilled water in a beaker and about 0.1 g of drug was added and stirred for 5 h and the drug content was determined as mentioned previously. For optimization of activation, resins were washed with distilled water and subsequently with 1N HCl. The resins were rewashed with water until neutral pH was reached. Resinates were prepared by dissolving 0.1 g of acid-activated resin in 100 ml distilled water containing 0.1 g of drug and stirred for 5 h and drug content was determined. Similarly, alkali activation of all resins were performed, replacing 1N HCl with 1N NaOH.

For optimization of pH, 0.1 g of drug was added to 0.1 g of activated resins in 100 ml of distilled water. The pH of solutions were adjusted to 3.0, 3.5, 4.0, 4.5, 5.0, 5.5, 6.0 and 6.5 and stirred for 5 h and the drug content was determined. For optimization of mode of mixing, rotary shaker and magnetic stirrer were used. All activated resins (0.1 g) in 100 ml of distilled water and about 0.1 g of drug. The pH was adjusted at 3.5 and drug content was determined. For ratio of drug:resin, three batches were prepared containing drug-resin in the ratio of 1:1, 1:2, 1:3. The pH was maintained at 3.5. The solution was stirred for 5 h. For optimization of temperature, 0.1 g of drug was added to 0.1 g of activated resins in 100 ml of distilled water. The pH was maintained at 3.5 and was stirred at 30°, 35°, 40°, 45°, 50°, 60°, 70° and the drug content was determined.

### Evaluation of micromeritic properties of resinates:

The different micromeritic properties of resinates like shape, flow properties, bulk density, tap density, packing ability were studied[12].

### Characterization of resinate:

FT-IR spectrum of the drug, resin and resinate were recorded over the wave no 4000 to 400 cm^-1^ on Jasco Dispersive type FT-IR spectrophotometer using the KBr disc technique. Then the spectra were analyzed to determine the formation of complex of drug and resin. The drug, resins and resinate was subjected to X-ray diffraction study for the confirmation of complex formation. X-ray diffraction studies were carried out on Phillips analytical X-ray BV (pw1710) using Cu anode 40 kv voltage and 30 mA current.

### Taste evaluation of solid drug:resin complex:

Drug resin complex (1:1) was subjected to sensory evaluation by a panel of nine members using time intensity method. Sample equivalent to 0.3 g of drug was held in mouth for 10 s. Bitterness was recorded instantly and then after 20, 30, 40, 50 and 60 min. The evaluation was performed by classifying bitter taste into five levels, level 0: no bitter taste is sensed, 1: acceptable bitterness, 2: slightly bitter, 3: moderately bitter, 4: strongly bitterness. Descriptive statistics mean and standard deviation were calculated for all variables. Paired t test was applied using INSTAT software. Value p< 0.05 has been considered as statistical significant level.

### Formulation of tablets:

Resinates of metformin hydrochloride (dose of drug 0.3 g) were formulated into tablet by wet granulation technique. Required quantity of resinate, HPMC (K100M) and MCC were blended in geometric fashion. Deionised water was added to powder blend and dispersed thoroughly in order to get the wet mass. The damp mass was shifted through sieve No-22 to obtain granules. The granules obtained were lubricated and compressed into tablet on tablet machine (Table 1).

**TABLE 1:** FORMULATION DESIGN

Ingredients	Formulations					
	B-1	B-2	B-3	B-4	B-5	B-6
Indion 244 Resinate	494*	494	494	-	-	-
Indion 264 Resinate	-	-	-	542*	542	542
MCC (PH 102)	84	72	60	36	24	12
HPMC (K100M)	48	60	72	48	60	72
Mg. Stearate	12	12	12	12	12	12
Talc	12	12	12	12	12	12
Total	650	650	650	650	650	650

* Containing 300 mg of drug

### Evaluation of tablets[13,14]:

Tablets were evaluated for various official and nonofficial specifications. Thickness was measured with the help of Vernier Caliper. The hardness of the tablets was measured with Monsanto hardness tester. For drug content uniformity, 20 tablets were weight and crushed. An accurately weighed 0.01 g drug equivalent resinates and transferred to 100 ml of 0.1 N HCl. This suspension was stirred on a magnetic stirrer for 5 h. The suspension was then filtered and the drug content was determined at 233.5 nm by making suitable dilutions.

### In vitro release studies:

Tablets formulated with resinates were subjected to *in vitro* dissolution studies using USP type II apparatus (paddle type) at 100 rpm with temperature of 37±0.5°. Dissolution was carried in 900 ml simulated gastric fluid for 2 h and for further 8 h in simulated intestinal fluid[15]. After 1 h interval, 5 ml dissolution medium was withdrawn by pipette. The samples withdrawn were diluted to 50 ml with buffer and filtered. The filtered samples were analyzed at 233.5 nm. The drug release data of Batch B-6 was fitted in various release kinetic equations such as Zero order, First order, Matrix, Peppas, Hix. Crow.

## RESULTS AND DISCUSSION

Metformin hydrochloride was loaded on ion exchange resin by batch process. Complexation is essentially a process of diffusion of ions between the resin and surrounding drug solution. As reaction is equilibrium phenomenon, maximum efficacy is best achieved in batch process. Complexation between drug and resin was found to be optimum after 5 h of mixing in all the resins investigated. Highest drug binding on resin was achieved when activated with 1N HCl. The drug loading was found to be 28.60±1.09, 25.32±0.49 for indion 244 and 264, respectively. After activation with acid treatment, the exchangeable ion on the resin is H^+^. Relative selectivity of H^+^ is least than other ionic form and therefore it increases percent complexation. Maximum drug loading on the resin occurs at pH 3.5; a maximum of 35.57±0.47, 29.75±1.16 for indion 244 and 264, respectively. As pH increases above 3.5, percentage of drug loading decreases. This may be due to fact that the fraction of metformin hydrochloride (pK_a_ 11.5) protonation decreases as the pH increases and reduces the interaction with the resin[16].

Complexation was found to be optimum in case of stirring, a maximum of 35.57±1.23, 29.75±0.64 for indion 244 and 264 and in case of shaking 31.48±0.92, 24.67±0.86 for indion 244 and 264 respectively. This finding may indicate the significant involvement of van der waals forces taking place along with drug exchange during complexation. Drug resin in the ratio of 1:1 gives optimum loading. The drug loading was found to be 35.57±0.64, 29.75±0.35 for indion 244 and 264, respectively. Increase in the amount of resin increases the amount of drug adsorbed from the solution but decreases the drug content per 100 mg of resinates. Maximum drug loading on the resin occurs at a temperature of 60°; a maximum of 62.30±1.31, 56.18±0.74 for indion 244 and 264, respectively. Increased temperature during complexation increases ionization of drug and resin. Higher temperatures tend to increase the diffusion rate of ions by decreasing the thickness of exhaustive exchange zone.

The different micromeritic properties resinates like shape, flow properties, bulk density, tap density, packing ability were studied (Table 2).The results showed that the resinates have good flow properties and packing abilities.

**TABLE 2:** MICROMERITIC PROPERTIES OF RESINATES*

Character	Indion 244 resinate	Indion 264 resinate
Shape	Irregular	Irregular
Angle of repose	32.65	0.3233
Bulk density (gm/cm^3^)	0.7142	0.6944
Tap density (g/cm^3^)	0.7575	0.7352
Carr's index (%cc)	5.716	0.5549
Housner ratio	1.06	1.058

* Average of three determinations

**Fig. 1:**
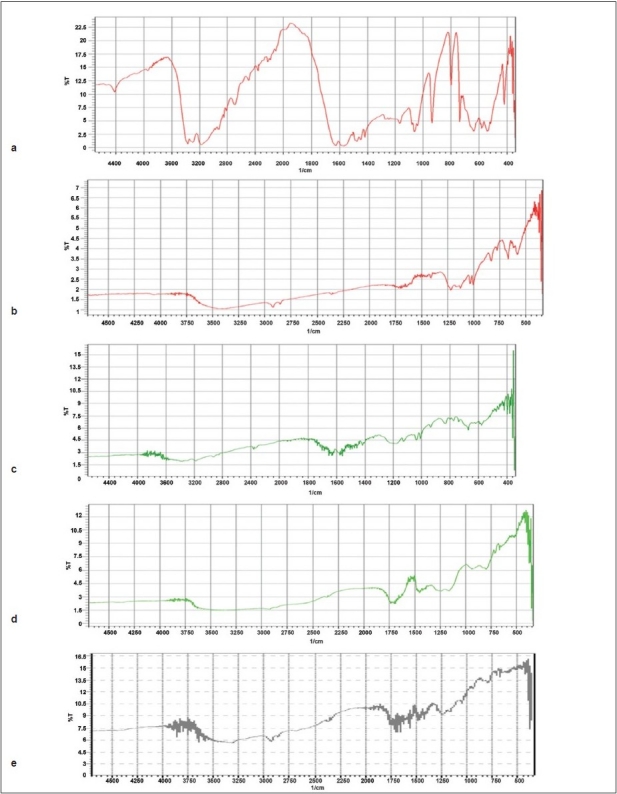
FT-IR spectra of metformin and resinates FT-IR spectra of (a) metformin hydrochloride, (b) Indion 244 resin, (c) Indion-244 resinate, (d) Indion 264 resin and (e) Indion-264 resinate

**Fig. 2:**
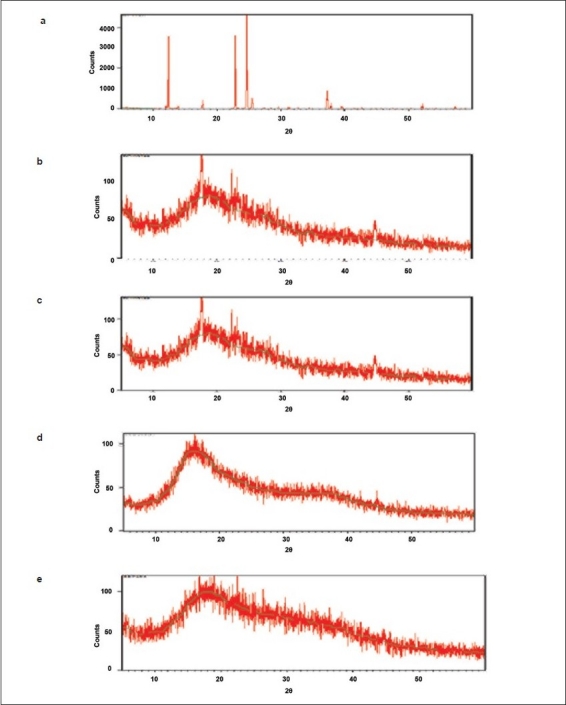
X-ray diffraction pattern of metformin and its resinates X-ray diffraction pattern of (a) Metformin hydrochloride, (b) Indion 244 resin, (c) Indion-244 resinate, (d) Indion 264 resin and (e) Indion-264 resinate

The infrared spectra of drug, indion 244 and 264 resins and resinate are depicted in fig. 1. FT-IR spectra of drug shows peak at 1028 cm^−1^ corresponding to the NH stretching in a secondary amine. Indion 244 and 264 shows characteristic peaks at 1674 cm^−1^ and 1722 cm^−1^ corresponding to −C=O stretching of aryl acids and due to aromatic C=C stretching. The absence of peak at 1028 cm ^−1^ in DRC confirms the complexation of the secondary amine group in the drug with resin. The X-Ray Diffraction study of drug shows highly crystalline nature. Resins indion 244 and indion 264 showed amorphous nature and the resinates showed noncrystalline characteristics. This might be because of entrapment of drug molecule in the polymer matrix of the resins. From all the evidences it can be concluded that the drug resinate was a chemical complex (fig. 2). Studies have shown that the molecules of the entrapped drug changes from crystalline to amorphous state[17].

Panel of 9 members using time intensity method determined the threshold bitterness value. Taste evaluation in volunteers confirmed that the taste of drug was masked by complexing with Indion resin. The majority of the volunteers found the DRC to be tasteless and agreeable (Table 3).

**TABLE 3:** VOLUNTEERS OPINION TEST FOR METFORMIN HCL BEFORE AND AFTER TASTE MASKING

Time (sec)	Before taste masking Mean±SD	After taste masking with Indion 244 Mean±SD	After taste Indion 264 masking with Mean±SD
10	4.0±0.00**	0.3±0.44**	0.2±0.44**
20	3.3±0.50**	0.2±0.33**	0.1±0.33**
30	2.55±0.52**	0	0
40	2.0±0.50**	0	0
50	1.77±0.44**	0	0
60	1.22±0.44**	0	0

** *P* < 0.0001

The prepared batches (B1-B6) of tablets were evaluated for various official and non-official parameters. Tablets were obtained of uniform weight due to uniform die fill with acceptable variations as per IP specifications, i.e. below 7.5%. The hardness of tablets for each formulation was between 4-5 kg/cm^2^. Average thickness was found to be in the range of 7 to 8 mm. Friability below 1% was an indication of good mechanical resistance of the tablets. The uniformity of drug content was found to be 97%-99% w/w which was within acceptable limits. Results are shown in Table 4.

**TABLE 4:** EVALUATION OF PHYSICAL CHARACTERISTICS OF TABLETS

Evaluation parameters	Formulations					
	B-1	B-2	B-3	B-4	B-5	B-6
Hardness (kg)	4.14±0.46	4.25±0.85	4.38±1.32	4.21±0.68	4.41±0.32	4.53±0.35
Thickness (mm)	7.66±0.13	7.55±0.63	7.40±0.85	7.53±0.93	7.26±0.35	7.21±0.46
Friability (%w/w)	0.66	0.63	0.58	0.54	0.49	0.44
Drug content (%w/w)	97.33	98	98.33	98.1	97.22	99
% Weight variation	2.45±0.4	1.85±0.4	1.45±0.3	2.08±1.6	2.78±0.5	1.23±0.2

**Fig. 3:**
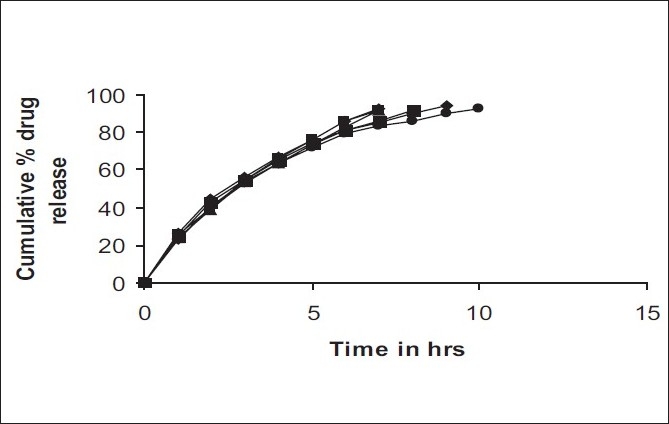
Cumulative % of drug release from tablets Cumulative percent drug release from tablets of batches B-1 (–□–); B-2 (–■–); B-3 (–▲–); B-4 (–■–); B-5 (–■–); B-6 (–●–)

Results of the *in vitro* release studies of various formulations designed and manufactured are presented in (Table 5) and are shown in fig. 3. The result showed that, in case of indion 244 resinate tablet, more than 92% of drug released from tablets formulation with HPMC (K100M) in 8 to 10% concentration within 7 h (Batch B1, B2). Addition of 12% HPMC (K100M) does not affect the drug release significantly (Batch B3). This may be due to rapid disintegration of tablets in dissolution medium because of larger particle size of Indion 244 resinate. In case of Indion 264 resinate tablets (Batch B-4, B-5) with 8-10% HPMC, more than 93% of the drug released within 8-9 h. Addition of 12% HPMC, more than 92% of drug released from tablet for 9 to 10 h (Batch B-6). This may be because of strong binding properties of HPMC which binds the fine particles of resinate. The drug release from these tablets was simply due to slow erosion and ion exchange. Tablets thus formulated with Indion 264 and 12% HPMC (K100M) provided sustained release of drug over a period of time of 10 h. The release of metformin hydrochloride from resinate controls the diffusion of drug molecules through the polymeric material into aqueous medium. The B-6 formulation shows precise sustained release of metfomin hydrochloride and follows first order kinetics with correlation coefficient of 0.999. Hence it was concluded that, formulation B-6 was selected as best formulation.

**TABLE 5:** CUMULATIVE % OF DRUG RELEASE FROM TABLETS

Time (h)	Dissolution medium	Cumulative drug release (Mean±SD, n=3)					
		B-1	B-2	B-3	B-4	B-5	B-6
1	1.2 PH Buffer	26.23±0.74	25.87±0.73	23.96±0.50	24.55±0.59	23.32±2.48	22.73±0.47
2	1.2 PH Buffer	44.87±1.63	39.30±0.78	38.53±1.48	42.95±2.48	40.88±1.48	39.56±0.48
3	6.8 PH Buffer	56.6±1.43	54.2±0.52	53.55±1.59	54.82±0.48	53.52±0.48	53.3±0.48
4	6.8 PH Buffer	66.62±0.57	65.90±0.95	63.89±0.48	64.99±1.39	63.88±0.49	63.79±1.49
5	6.8 PH Buffer	76.23±0.73	75.87±0.59	73.61±0.58	74.63±0.49	73.73±0.95	71.97±1.49
6	6.8 PH Buffer	85.56±1.72	85.68±0.59	83.00±0.90	81.12±0.49	80.92±0.83	79.16±0.49
7	6.8 PH Buffer	92.63±1.16	91.98±1.48	91.52±1.49	86.12±0.37	84.91±1.49	83.18±0.49
8	6.8 Ph Buffer				91.96±1.40	89.74±2.54	85.96±0.58
9	6.8 PH Buffer					93.86±1.48	89.75±1.46
10	6.8 PH Buffer						92.37±1.47

Figures of % drug release are mean of triplicate study
